# Association between Life’s Crucial 9 and bowel health among US adults: a cross-sectional analysis of NHANES 2005–2010 with external validation

**DOI:** 10.3389/fmed.2025.1687499

**Published:** 2025-10-31

**Authors:** Xueman Wang, Jian Dong, Bo Lian, Xintong Jiang

**Affiliations:** Department of Endoscopy Center, Shaoxing People’s Hospital (The First Affiliated Hospital, Shaoxing University), Shaoxing, Zhejiang, China

**Keywords:** Life’s Crucial 9, diarrhea, constipation, NHANES, cardiovascular health

## Abstract

**Background:**

Abnormal bowel health, including constipation and diarrhea, is common in the general population. The American Heart Association recently introduced Life’s Essential 8 (LE8) as a framework for assessing cardiovascular health (CVH). Building on this, Life’s Crucial 9 (LC9) was developed by adding psychological health as a ninth component, providing a more holistic measure of overall health. Our objective was to investigate whether LC9 is linked to bowel health. To our knowledge, this is the first study to examine the association between LC9 and bowel health outcomes using nationally representative data, with validation in an independent hospital-based cohort.

**Methods:**

This cross-sectional analysis included 12,817 adults from Nutrition Examination Survey (NHANES) 2005–2010, and validation was conducted in an external hospital-based cohort of 991 patients. The primary exposure was the LC9 score, and the primary outcomes were constipation and diarrhea. The primary analysis used weighted multivariable logistic regression to assess the associations of LC9 scores with bowel outcomes. Restricted cubic splines (RCS) were applied to evaluate potential non-linear relationships. Sensitivity and stratified analyses were performed to test the stability of the results. Internal validation was conducted within the U.S. NHANES cohort, and external validation was performed using a hospital-based cohort from our center to confirm robustness and generalizability.

**Results:**

After adjusting for potential confounders, higher LC9 scores were significantly associated with a lower risk of both constipation [adjusted odds ratio (AOR) = 0.90, *p* = 0.001] and diarrhea (AOR = 0.82, *p* < 0.001). Compared to participants in the lowest tertile, those in the highest LC9 tertile showed a reduced risk of constipation (AOR = 0.70, *p* < 0.001) and diarrhea (AOR = 0.54, *p* < 0.001). RCS analysis demonstrated a linear dose–response relationship between LC9 and bowel health outcomes. Stratified analyses confirmed consistent associations across most subgroups, with no significant interactions except for race in the LC9-diarrhea relationship. Sensitivity analyses using unweighted logistic models yielded similar results. Validation analyses confirmed consistent performance, with good discrimination, calibration, and net clinical benefit across cohorts.

**Conclusion:**

A negative linear association was found between LC9 and both constipation and diarrhea in US adults. Future longitudinal cohort studies are needed to assess the association between LC9 and bowel health.

## 1 Introduction

Constipation and diarrhea are among the most common gastrointestinal complaints worldwide and represent a substantial public health burden (1). Epidemiological studies have shown that approximately 14–20% of adults worldwide experience chronic bowel symptoms (2, 3). In the United States, approximately 16% of adults report constipation, and the prevalence increases to nearly one-third among individuals aged 60 years and older (4). These conditions not only cause discomfort and impaired quality of life but are also linked to psychological distress, reduced work productivity, and increased healthcare costs (5). In the U.S. alone, millions of outpatient visits annually are attributable to bowel disorders, highlighting their significant societal impact (6, 7). Importantly, bowel disturbances often coexist with cardiometabolic risk factors and mental health conditions, further amplifying their clinical relevance (8, 9). Despite their clinical significance, bowel symptoms are frequently overlooked and insufficiently addressed in routine clinical care (10, 11).

A growing body of literature has highlighted the relevance of systemic health—especially cardiovascular health (CVH)—in the pathogenesis and progression of gastrointestinal (GI) symptoms. Both the cardiovascular and GI systems are closely linked through shared inflammatory, metabolic, and neurohormonal pathways (12–15). Chronic systemic inflammation, for example, is a well-established risk factor for cardiovascular disease (CVD) and has also been implicated in altered bowel function (16, 17). Similarly, dysbiosis of the gut microbiota, commonly observed in individuals with unhealthy lifestyles or CVD, may contribute to disrupted gut barrier integrity and abnormal bowel habits (18, 19). Furthermore, short-chain fatty acids (SCFAs) derived from dietary fiber fermentation play a protective role in both gut and cardiovascular homeostasis through their anti-inflammatory and lipid-modulating properties (20).

In 2022, the American Heart Association proposed the Life’s Essential 8 (LE8), an updated framework for quantifying CVH. It integrates four behavioral metrics—diet, physical activity, smoking status, and sleep health—and four physiological metrics—body mass index (BMI), blood pressure, fasting glucose, and cholesterol levels (21). Building on this foundation, an extended framework called Life’s Crucial 9 (LC9) was proposed, which adds psychological health [assessed by Patient Health Questionnaire-9 (PHQ-9)] as a ninth component to the original eight metrics of Life’s Essential 8 (LE8). Thus, LC9 integrates lifestyle, physiological, and psychological domains to provide a more comprehensive measure of overall health. The inclusion of psychological factors reflects the growing recognition of psychological well-being as a determinant of both cardiovascular and GI function (22). Mental health disorders such as anxiety and depression have been independently associated with altered bowel habits via the gut–brain axis, influencing motility, secretion, and visceral sensitivity (23, 24).

The Nutrition Examination Survey (NHANES) has been carried out every 2 years since 1999. Each year, a complicated multistage random sampling method is used to obtain a sample of roughly 5,000 patients. Data from a variety of laboratory and physical examinations, as well as standardized interview questions, are part of NHANES (25). NHANES is uniquely valuable for this study because it collects both lifestyle factors (diet, sleep, physical activity, smoking) and standardized clinical measures (BMI, blood pressure, glucose, lipids, PHQ-9), which allow for the comprehensive calculation of LC9. While these data provide robust insights into U.S. adults, it should be noted that differences in diet patterns, obesity prevalence, and mental health burden compared with other countries may limit generalizability, underscoring the need for future studies in non-U.S. populations.

Despite the conceptual relevance of LC9 to bowel health, no study to date has systematically examined its relationship with diarrhea and constipation in a nationally representative population. Given that constipation and diarrhea remain common yet under-recognized problems with substantial impact on quality of life and healthcare burden, there is a clear need for more holistic risk assessment tools. By incorporating psychological health into the traditional LE8 framework, LC9 provides a novel and integrative measure of overall health. The study settings provide unique advantages. NHANES is the only nationally representative survey in the United States that concurrently collects gastrointestinal symptom data, lifestyle behaviors, cardiometabolic indicators, and psychological health, which uniquely enables comprehensive calculation of LC9 and its direct linkage to bowel outcomes. To complement these population-based data, we further validated the findings in a hospital-based Chinese cohort from a tertiary endoscopy center, where bowel complaints are a leading reason for consultation. This dual-site design integrates community-level epidemiology with real-world clinical practice, thereby strengthening both the relevance and generalisability of our findings. Therefore, the objective of this study was to investigate the association between LC9 scores and bowel health status, specifically focusing on the prevalence of chronic diarrhea and constipation. To our knowledge, our study is novel in being the first to evaluate LC9 and bowel health (constipation and diarrhea) using nationally representative data, with external validation in an independent hospital-based cohort. By addressing this gap, our work contributes new evidence on the systemic role of holistic health, including psychological well-being, in bowel function.

## 2 Methods

### 2.1 Population and study design

**Figure 1:**
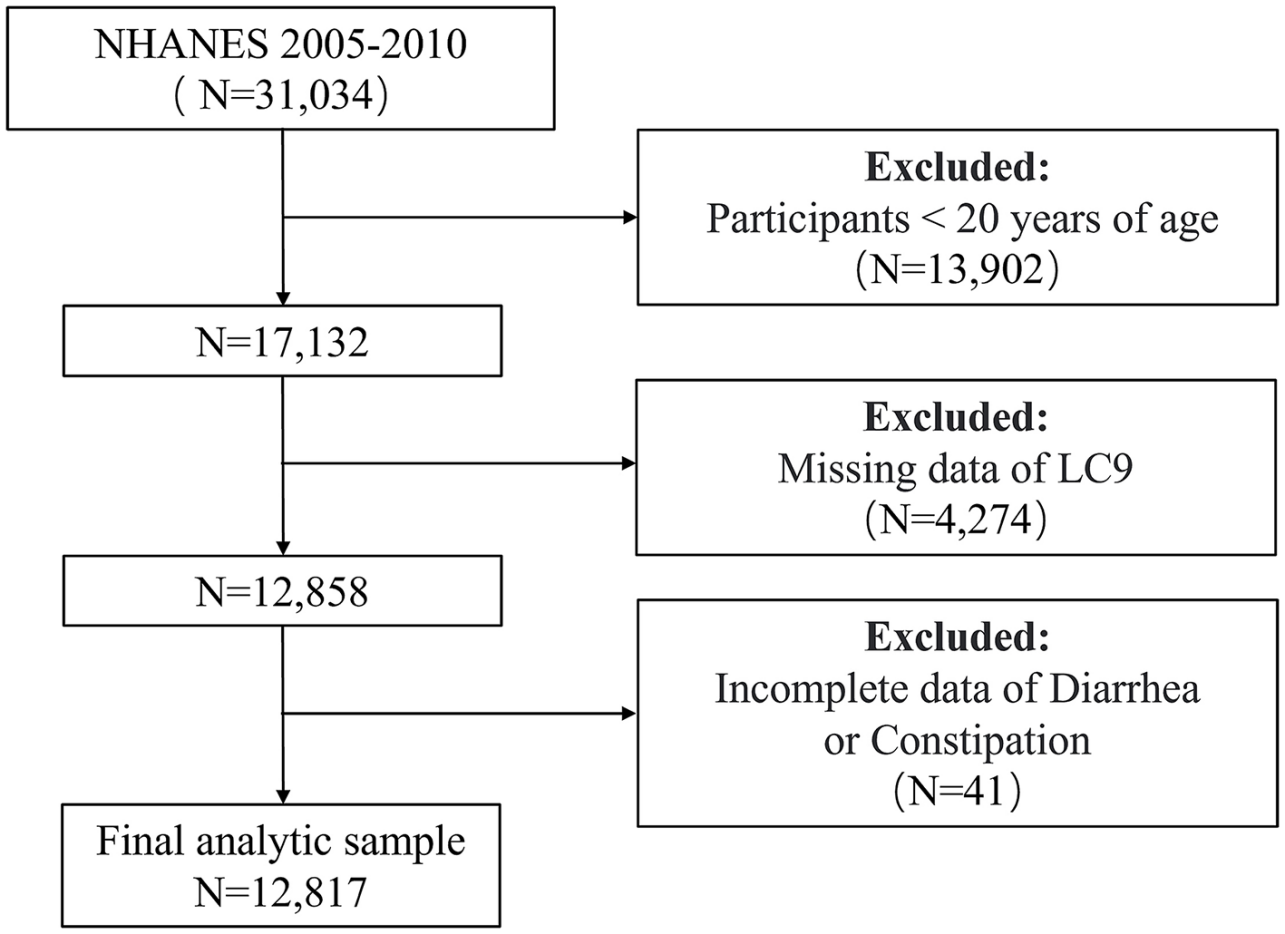
Flowchart for screening eligible participants in the NHANES 2005–2010 cross-sectional study. NHANES, National Health and Nutrition Examination Survey. LC9, Life’s Crucial 9.

This was a cross-sectional study based on data from the NHANES 2005–2010. Both exposures (LC9 scores) and outcomes (bowel health status) were assessed at the same survey wave, and associations were examined using weighted multivariable logistic regression. The primary exposure was the LC9 score, which was chosen because lifestyle, cardiometabolic, and psychological domains are all mechanistically linked to gastrointestinal function through systemic inflammation, gut microbiota composition, autonomic regulation, and the gut–brain axis (12, 26). The primary outcomes were constipation and diarrhea, defined by both stool frequency and stool consistency. These outcomes were selected because they are the two most common and burdensome bowel disturbances worldwide (2, 6). The NHANES protocols were approved by the NCHS Research Ethics Review Board, and written informed consent was obtained from all participants. We started by removing all individuals under the age of 20 (*n* = 13,902) from the 31,034 who took part in the NHANES 2005–2010 study. Participants whose LC9 (*n* = 4,274) and bowel health (*n* = 41) data was missing were subsequently removed. In the end, the following analysis comprised 12,817 individuals (Figure 1).

### 2.2 Assessment of LC9

LC9 is a composite score derived from the eight metrics of LE8 plus a rescaled Psychological Health Score (PHQ-9), with each component standardized to a 0–100 scale. The LE8 framework comprises four behavioral metrics—dietary intake, physical activity, tobacco exposure, and sleep duration—and four physiological indicators, including BMI, blood pressure, blood glucose, and serum lipid levels. Dietary intake was evaluated using data from a single 24-h dietary recall, scored using the Healthy Eating Index-2015 (HEI-2015) (27) (Supplementary Table 1). Self-reported responses from structured questionnaires were used to assess physical activity patterns, smoking status and sleep duration. Laboratory assessments and physical measurements were used to determine lipid profiles, glucose levels, anthropometric data, and blood pressure. The LC9 score was constructed by extending the LE8 framework with an additional psychological health component derived from the PHQ-9. The eight LE8 metrics were each scored on a scale of 0–100 following the American Heart Association guidelines. PHQ-9 scores (range: 0–27) were categorized into five severity levels: 0–4 (none/minimal), 5–9 (mild), 10–14 (moderate), 15–19 (moderately severe), and 20–27 (severe). These were then rescaled to 100, 75, 50, 25, and 0, respectively, to match the LE8 scoring system. The LC9 score was calculated as the arithmetic mean of all nine metrics, with equal weight assigned to each component. Based on the distribution of LC9 scores in the study population, participants were divided into three groups using tertiles (i.e., the 33rd and 66th percentiles) to ensure comparability across categories. T1 (lowest): LC9 scores <the 33rd percentile; T2 (middle): LC9 scores between the 33rd and 66th percentiles; T3 (highest): LC9 scores >the 66th percentile. A full description of the scoring procedures applied to NHANES data for LC9 is available in Supplementary Table 2.

### 2.3 Diagnosis of constipation and diarrhea

The NHANES database used two criteria for defining constipation and diarrhea: stool frequency and stool consistency. For 30 days prior to data collection, participants were asked to report the frequency and consistency of their feces.

The question that was used to measure stool frequency was, “How many times per week do you usually have a bowel movement?” Constipation was defined as a response of fewer than 3 times per week, whereas diarrhea was defined as an answer of 21 times per week or more. The Bristol Stool Form Scale (BSFS), which includes seven distinct forms of stool and a variety of colored cards with extensive explanations for each, was used to evaluate stool consistency (28). Type 1 (hard, nut-like lumps) and type 2 (lumpy, sausage-shaped stools) were classified as constipation, while type 6 (mushy, fluffy pieces with ragged edges) and type 7 (watery, entirely liquid stools) were classified as diarrhea. Stool types classified as BSFS 3 to 5, along with other reported bowel movement frequencies, were considered normal.

### 2.4 Covariates

For the purpose of controlling for confounders, a number of possible variables were included. Potential confounders were identified *a priori* based on prior literature demonstrating their associations with both lifestyle/psychological health and gastrointestinal outcomes (29, 30). The following demographic variables were included in our sociodemographic analysis: age, sex, race, educational level (divided into three groups based on high school boundaries), marital status (classification according to whether they are coupled or not) and poverty-income ratio (PIR) (<2, ≥2). All covariates were taken from the NHANES demographic and questionnaire datasets and were included in multivariable models to adjust for potential confounding.

### 2.5 External validation cohort

An independent hospital-based cohort was established at our hospital between January 2022 and March 2025, comprising 991 adult participants. Inclusion criteria were: (1) age ≥20 years; (2) complete information on all LC9 components, including lifestyle factors (diet, physical activity, smoking status, and sleep health), physiological indicators (BMI, blood pressure, blood glucose, and serum lipids), and mental health (PHQ-9); and (3) available bowel health information. Exclusion criteria included: (1) history of organic gastrointestinal diseases such as inflammatory bowel disease, colorectal cancer, or severe diverticulitis; (2) recent use (within the past 1 month) of medications known to affect bowel function (e.g., laxatives, opioids, anticholinergic drugs); (3) pregnancy or lactation. Baseline demographic and clinical characteristics of this cohort are presented in Supplementary Table 3. In the external validation cohort, bowel health was assessed using the same criteria as in NHANES to ensure comparability. Stool frequency and consistency were collected through standardized questionnaires administered during hospital visits. Constipation was defined as fewer than three bowel movements per week or BSFS types 1–2, whereas diarrhea was defined as 21 or more bowel movements per week or BSFS types 6–7. The hospital-based validation cohort was approved by the Ethics Committee of Shaoxing People’s Hospital, and informed consent was waived due to the retrospective design.

### 2.6 Statistical analysis

All analyses were suitably weighted in accordance with the NHANES Analytic Guidelines, and all data handling and analysis were conducted using the R program (version 4.2.3). Continuous variables were expressed as mean (standard error) and tested using weighted Kruskal–Wallis tests (three groups), and categorical variables were expressed as numbers (weighted percentage) and analyzed using the weighted chi-square test. The primary analytical approach was weighted multivariable logistic regression, conducted according to the NHANES Analytic Guidelines to account for the complex, multistage survey design. Odds ratios (ORs) and 95% confidence intervals (CIs) were estimated for the associations between LC9 scores and bowel health outcomes (constipation and diarrhea). No variables were accounted for in the Model 1. Model 2 took into account demographic factors such as sex, age, race. Model 3 also accounted for PIR, marital status, and level of education. To investigate nonlinear relationships or dose–response connections, restricted cubic spline (RCS) was used. Through interaction analyses, we were able to discover possible effect modifiers, and stratified analyses allowed us to investigate the stability of connections between various subgroups. In addition, in the sensitivity analysis, we did not weight the data and adopted multivariable logistic regression analysis to further verify the stability of the results. Taken together, to strengthen internal validity and reduce the potential influence of self-report bias, we relied on standardized instruments (e.g., BSFS visual cards, PHQ-9), incorporated stepwise adjustment for potential confounders in three multivariable logistic regression models, and conducted multiple robustness checks, encompassing stratified subgroup analyses, sensitivity analyses using unweighted models, and external validation in an independent hospital-based cohort.

To assess the predictive performance of the models, we randomly divided the NHANES participants into a training set and an internal validation set in a 7:3 ratio. An independent external cohort comprising 991 patients from our hospital between 2022 and 2025 was further used for validation. Discrimination was evaluated by receiver operating characteristic (ROC) curves and the area under the curve (AUC). Calibration was assessed using calibration plots generated by the bootstrap method. Decision curve analysis (DCA) was performed to determine the net clinical benefit across a range of threshold probabilities. A bilateral *p*-value <0.05 was considered statistically significant.

## 3 Results

### 3.1 Characteristics of the participants

The 12,817 people who took part in the research had an average age of 47.35 years, and women made up 52.3 percent of the sample. Among the participants, 1,331 (9.5%) had constipation, and 1,069 (7.3%) had diarrhea. Compared with the normal group, both the constipation and diarrhea groups had a higher proportion of females and non-Hispanic Black people, lower educational attainment, and were more likely to live in poverty and be not coupled (*p* < 0.001). The LC9 scores in the constipation and diarrhea groups were 67.37 and 64.95, respectively, both lower than that of the normal group (69.54) (*p* < 0.001). Similar patterns were observed in HEI-2015 diet score, physical activity, sleep health, and psychological health, all of which were significantly lower in the constipation and diarrhea groups (all *p* < 0.001). In addition, obesity, hyperlipidemia, hypertension, and diabetes mellitus were more common among participants with diarrhea. Details are presented in Table 1.

**Table 1:** Weighted characteristics of the study cohort.

Variables	Total (*n* = 12,817)	Constipation (*n* = 1,331)	Diarrhea (*n* = 1,069)	Normal (*n* = 10,417)	*p*
Age (years), mean (SE)	47.35 (0.35)	45.39 (0.49)	50.69 (0.72)	47.28 (0.37)	<0.001
Age (years), *n* (%)					<0.001
20–39	4,235 (35.6)	538 (41.0)	247 (27.1)	3,450 (35.7)	
40–59	4,192 (39.7)	412 (36.7)	371 (42.7)	3,409 (39.8)	
≥60	4,390 (24.7)	381 (22.3)	451 (30.3)	3,558 (24.5)	
Sex, *n* (%)					<0.001
Male	6,158 (47.7)	351 (23.3)	473 (44.8)	5,334 (50.8)	
Female	6,659 (52.3)	980 (76.7)	596 (55.2)	5,083 (49.2)	
Race, *n* (%)					<0.001
Mexican American	2,261 (7.7)	217 (7.8)	226 (9.4)	1,818 (7.5)	
Other Hispanic	1,057 (4.2)	128 (5.2)	102 (4.8)	827 (4.1)	
Non-Hispanic White	6,521 (72.3)	595 (65.8)	480 (68.1)	5,446 (73.5)	
Non-Hispanic Black	2,495 (10.6)	345 (16.4)	216 (11.7)	1,934 (9.8)	
Other races	483 (5.2)	46 (4.8)	45 (6.0)	392 (5.1)	
Educational level, *n* (%)					<0.001
Below high school	3,440 (17.3)	413 (21.5)	406 (25.0)	2,621 (16.1)	
High school	3,076 (24.3)	368 (29.5)	250 (24.7)	2,458 (23.7)	
Above high school	6,301 (58.4)	550 (49.0)	413 (50.3)	5,338 (60.2)	
PIR, *n* (%)					<0.001
<2	5,353 (29.8)	662 (39.9)	522 (34.5)	4,169 (28.2)	
≥2	6,558 (64.7)	563 (53.4)	457 (59.2)	5,538 (66.5)	
Not record	906 (5.5)	106 (6.7)	90 (6.3)	710 (5.3)	
Marital status, *n* (%)					0.001
Coupled	7,993 (66.1)	757 (60.3)	666 (66.4)	6,570 (66.8)	
Not coupled	4,824 (33.9)	574 (39.7)	403 (33.6)	3,847 (33.2)	
LC9 score, mean (SE)	69.00 (0.32)	67.37 (0.60)	64.95 (0.57)	69.54 (0.33)	<0.001
HEI-2015 diet score, mean (SE)	38.63 (0.58)	33.86 (0.98)	36.72 (1.13)	39.34 (0.63)	<0.001
Physical activity score, mean (SE)	67.17 (0.76)	60.70 (1.36)	62.26 (1.51)	68.35 (0.80)	<0.001
Nicotine exposure score, mean (95% CI)	69.34 (0.71)	69.00 (1.91)	65.07 (1.68)	69.76 (0.68)	0.002
Sleep health score, mean (SE)	82.35 (0.37)	78.63 (0.99)	79.79 (0.92)	83.00 (0.35)	<0.001
BMI score, mean (SE)	61.94 (0.57)	66.23 (1.35)	54.42 (1.24)	62.11 (0.60)	<0.001
Blood lipids score, mean (SE)	61.92 (0.40)	63.67 (1.08)	58.29 (1.30)	62.05 (0.42)	0.009
Blood glucose score, mean (SE)	87.32 (0.31)	87.21 (0.63)	83.15 (1.01)	87.70 (0.32)	<0.001
Blood pressure score, mean (SE)	70.13 (0.48)	73.92 (0.89)	65.91 (1.21)	70.07 (0.51)	<0.001
Psychological health score, mean (SE)	91.94 (0.28)	86.93 (0.78)	86.22 (1.06)	93.02 (0.27)	<0.001

LC9, Life’s Crucial 9; PIR, poverty income ratio; BMI, body mass index; HEI-2015, Healthy Eating Index-2015; SE, standard error.

### 3.2 Association between LC9 and constipation

**Figure 2:**
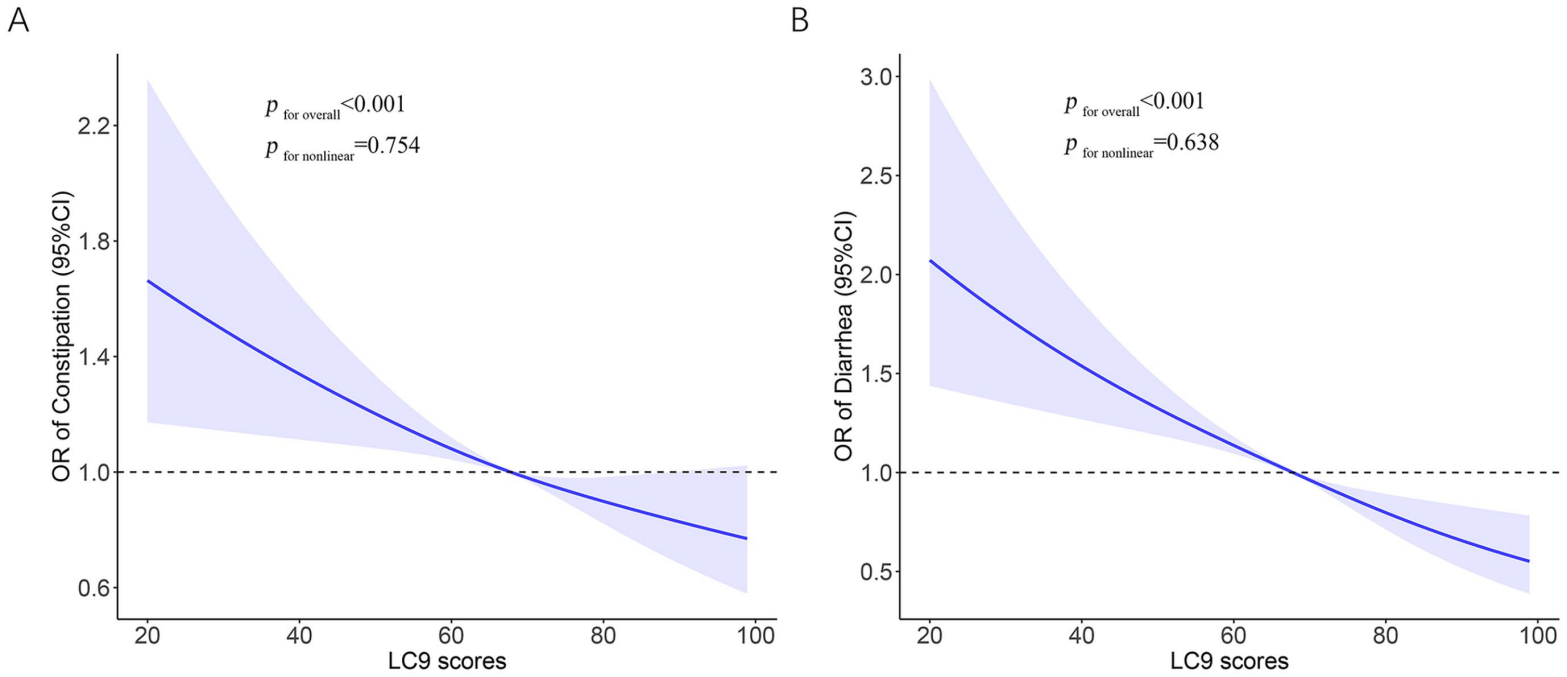
Restricted cubic spline analysis with multivariate-adjusted to identify the relationship between LC9 and bowel health. **(A)** LC9 and constipation. **(B)** LC9 and diarrhea. LC9, Life’s Crucial 9; OR, odds ratios; CI, confidence interval.

There was a link between LC9 and constipation, as shown in Table 2 of the logistic regression modeling findings. Unadjusted logistic regression demonstrated a negative connection between LC9 scores and constipation, with an OR of 0.89 (95% CI: 0.84–0.94). There was still a strong negative correlation between LC9 scores and constipation in Model 3 [adjusted odds ratio (AOR) = 0.90, 95% CI: 0.85–0.96]. The results demonstrated that after dividing the LC9 scores into three groups based on tertiles, the AOR for constipation was lower in the middle (T2: AOR = 0.81) and highest (T3: AOR = 0.70) groups compared to the lowest group (T1), and this was also supported by the trend test (*p* < 0.05). For this study, we analyzed the LC9-constipation association using RCS. These findings pointed to a linear negative association between LC9 scores and constipation risk (*p* for nonlinear = 0.754) (Figure 2A).

**Table 2:** Weighted logistic regression analysis of the correlation between LC9 scores and the prevalence of constipation and diarrhea.

Variables	Model 1		Model 2		Model 3	
	OR (95% CI)	*p*	OR (95% CI)	*p*	OR (95% CI)	*p*
Constipation						
LC9 scores (per 10 points)	0.89 (0.84, 0.94)	<0.001	0.86 (0.81, 0.91)	<0.001	0.90 (0.85, 0.96)	0.001
Tertile						
T1	Ref.		Ref.		Ref.	
T2	0.74 (0.64, 0.85)	<0.001	0.74 (0.63, 0.86)	<0.001	0.81 (0.69, 0.94)	0.007
T3	0.69 (0.57, 0.83)	<0.001	0.59 (0.48, 0.72)	<0.001	0.70 (0.57, 0.85)	<0.001
*p* for trend	<0.001		<0.001		<0.001	
Diarrhea						
LC9 scores (per 10 points)	0.79 (0.74, 0.84)	<0.001	0.80 (0.75, 0.85)	<0.001	0.82 (0.77, 0.88)	<0.001
Tertile						
T1	Ref.		Ref.		Ref.	
T2	0.60 (0.51, 0.72)	<0.001	0.62 (0.53, 0.74)	<0.001	0.64 (0.55, 0.76)	<0.001
T3	0.46 (0.38, 0.56)	<0.001	0.48 (0.39, 0.59)	<0.001	0.54 (0.43, 0.66)	<0.001
*p* for trend	<0.001		<0.001		<0.001	

Model 1, not adjusted; Model 2, adjusted for age (continuous), sex, race; Model 3, additionally adjusted for educational level, PIR and marital status. LC9, Life’s Crucial 9; OR, odds ratios; CI, confidence interval.

### 3.3 Association between LC9 and diarrhea

An inverse relationship between LC9 scores and the prevalence of diarrhea was also observed. Individuals with higher LC9 scores were less likely to report diarrhea, with a fully AOR of 0.82 (95% CI: 0.77–0.88). When LC9 was analyzed in tertiles, both the middle (T2) and highest (T3) groups showed significantly reduced odds of diarrhea compared to the lowest group (T1), with AORs of 0.64 and 0.54, respectively. A clear dose–response trend was detected (*p* for trend<0.05) (Table 2). The RCS model further supported a linear inverse association (*p* for nonlinear = 0.638), as illustrated in Figure 2B.

### 3.4 Stratified analysis

**Figure 3:**
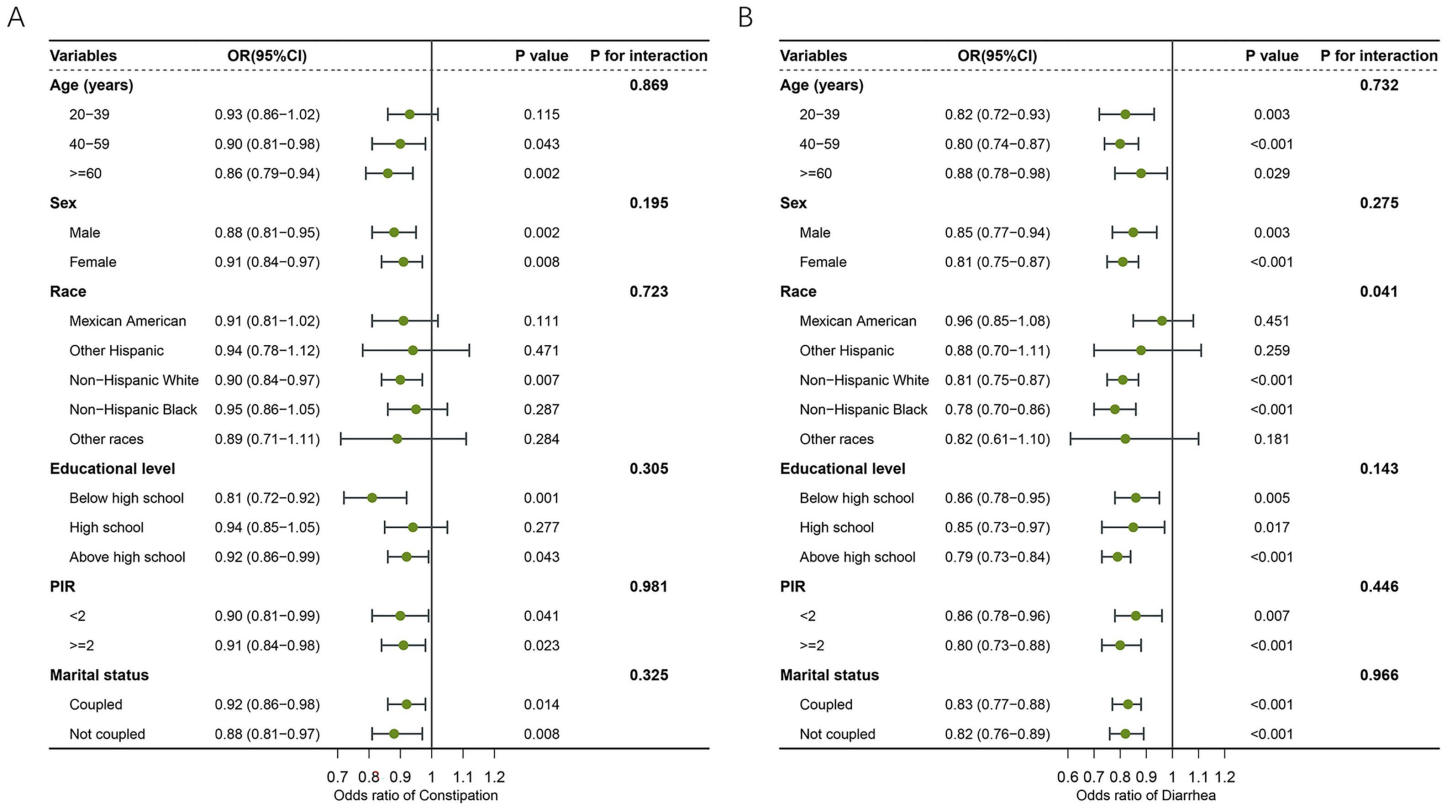
Forest plot illustrating the subgroup level of association between LC9 and bowel health. **(A)** LC9 and constipation. **(B)** LC9 and diarrhea. LC9, Life’s Crucial 9; OR, odds ratios; CI, confidence interval; PIR, poverty income ratio.

Stratified analyses were conducted to examine the association between LC9 scores and bowel symptoms across different subpopulations (Figure 3). For constipation (Figure 3A), LC9 was significantly associated with a lower risk of constipation in most strata, including both sexes, individuals aged ≥40 years, non-Hispanic white people, those with education below or above high school, and across all income and marital status groups (*p* < 0.05). All interaction tests were non-significant (*p* for interaction>0.05), suggesting a consistent association across subgroups. For diarrhea (Figure 3B), a similar inverse relationship was observed across most strata. The association remained significant regardless of sex, age, education, income level, and marital status. Notably, the association was also significant among non-Hispanic white people and non-Hispanic Black people, but not among Mexican Americans or other Hispanic people. The interaction test for race reached statistical significance (*p* = 0.041), indicating a potential modifying effect of race on the LC9–diarrhea relationship. However, other subgroup interactions were not significant.

### 3.5 Sensitivity analysis

According to the sensitivity analysis using unweighted logistic regression, in the fully adjusted model, LC9 scores remained negatively associated with both constipation and diarrhea. Similarly, individuals in the highest (T3) group had a lower risk of both constipation (AOR = 0.75, 95% CI: 0.65–0.88) and diarrhea (AOR = 0.61, 95% CI: 0.51–0.72) compared to those in the lowest group (T1) (Table 3). These results indicate that our findings are stable and robust.

**Table 3:** Unweighted logistic regression analysis of the correlation between LC9 scores and the prevalence of constipation and diarrhea.

Variables	OR	95% CI	*p*
Constipation			
LC9 scores (per 10 points)	0.92	0.88–0.96	<0.001
Tertile			
T1		Ref.	
T2	0.84	0.73–0.97	0.014
T3	0.75	0.65–0.88	<0.001
*p* for trend		<0.001	
Diarrhea			
LC9 scores (per 10 points)	0.85	0.81–0.89	<0.001
Tertile			
T1		Ref.	
T2	0.77	0.66–0.89	0.001
T3	0.61	0.51–0.72	<0.001
*p* for trend		<0.001	

The association was adjusted for age (continuous), sex, race, educational level, PIR, marital status. LC9, Life’s Crucial 9; OR, odds ratios; CI, confidence interval.

### 3.6 External validation of the predictive model

In the primary analysis, participants from the NHANES database were randomly divided into a training set and an internal validation set in a 7:3 ratio. To further examine the robustness and generalizability of the model, an external validation cohort consisting of 991 patients from our hospital between 2022 and 2025 was included. After evaluation, age, sex, educational level, marital status, and LC9 score were retained as significant variables in the final model for predicting constipation and diarrhea outcomes (Supplementary Table 3). Within the group of individuals with constipation, the calibration assessment demonstrated good agreement between predicted and actual outcomes in the external dataset. Furthermore, the external cohort achieved an AUC of 0.694 (95% CI: 0.655–0.734), which exceeded the performance observed in the training cohort (AUC = 0.671, 95% CI: 0.653–0.689) and the internal validation cohort (AUC = 0.668, 95% CI: 0.641–0.694), demonstrating the model’s robust discriminative ability and its potential generalizability to independent populations. Importantly, DCA revealed a clear net clinical benefit for the model when the threshold probability ranged from 0.15 to 0.65, indicating meaningful utility in guiding clinical decision-making (Supplementary Figure 1). In the diarrhea group, the model retained stable discrimination, with the external validation cohort yielding an AUC of 0.708 (95% CI: 0.669–0.746), higher than the training (AUC = 0.624, 95% CI: 0.603–0.644) and internal validation (AUC = 0.640, 95% CI: 0.608–0.672) sets. Calibration confirmed close agreement between predicted and observed outcomes, and DCA indicated a net clinical benefit when the threshold probability was between 0.18 and 0.67 (Supplementary Figure 2).

## 4 Discussion

Using a large and nationally representative sample from the NHANES database, we investigated the potential relationship between LC9 scores and bowel health, specifically focusing on constipation and diarrhea. Our findings revealed that higher LC9 scores were significantly associated with a reduced risk of both constipation and diarrhea. These associations remained robust even after adjusting for a range of sociodemographic and clinical covariates, underscoring the potential role of LC9 as a holistic predictor of GI well-being. The inverse linear trend between LC9 scores and bowel dysfunction was further supported by RCS analyses, suggesting a dose–response relationship. These results highlight the broader systemic impact of lifestyle and mental health factors, as encompassed by LC9, on bowel health.

Prior research based on the LE8 framework has identified important associations between CVH and bowel conditions (29–31). For example, Wang and Wang (29) reported that higher LE8 scores were significantly associated with a lower risk of diarrhea in a representative U.S. population. However, their study did not find a statistically significant association between LE8 scores and constipation. This suggests that while LE8 may capture certain aspects of bowel health, it may not fully account for the broader range of functional bowel disorders. In contrast, our study demonstrates that the more comprehensive LC9 framework, which includes depressive symptoms as an additional component, is significantly associated with reduced risks of both constipation and diarrhea. This suggests that incorporating mental health into the CVH model may enhance its ability to predict bowel dysfunction. As LC9 is a relatively new construct, it may be less familiar to some readers; however, it can be understood simply as LE8 plus a mental health component. Depression is known to influence bowel health through multiple mechanisms, including impaired intestinal motility, altered visceral sensitivity, dysbiosis, and increased inflammatory responses (23, 32). These pathophysiological pathways are more relevant to constipation than diarrhea in some populations, which may explain why LC9, but not LE8, captures this association. These findings emphasize the value of using LC9 as a more integrative and sensitive tool for assessing systemic determinants of bowel health in the general population.

Our study aligns with a growing body of evidence suggesting that healthy lifestyle behaviors, including a balanced diet, regular physical activity, sufficient sleep, and non-smoking status, play essential roles in maintaining GI function. These components, which are comprehensively captured in the LC9 framework, influence bowel health through multiple interrelated biological pathways. A diet rich in antioxidants and fiber, highlighted by the LC9 dietary metric, has been linked to improved gut mucosal integrity and greater microbial diversity (33). Gut microbiota ferment fibers and polyphenols into SCFAs such as butyrate, acetate, and propionate. These metabolites nourish colonocytes, regulate immune responses, and enhance intestinal motility, preventing intestinal disorders such as constipation and diarrhea (34). Physical activity is another important component that contributes to GI homeostasis. A recent meta-analysis of cohort studies confirmed that higher levels of physical activity significantly reduce the risk of constipation (35). Regular exercise has been associated not only with faster colonic transit time, improved autonomic regulation, and reduced systemic inflammation, but also with favorable modulation of the gut microbiota (36–38). Cigarette smoking has been associated with increased intestinal permeability, disruption of gut microbial balance, and elevated risk of GI disorders (39). Multiple studies have shown that smoking alters gut microbiota composition, favoring expansion of opportunistic pathogens and reducing beneficial microbes, while increasing mucosal permeability and impairing tight junction integrity (40, 41). These changes are accompanied by chronic low-grade inflammation, which may compromise gut motility and epithelial barrier function. Adequate sleep is essential for maintaining gut–brain axis stability (42). Disrupted sleep or circadian misalignment can alter gut microbiota composition, increase gut permeability, and elevate proinflammatory cytokines. Short sleep duration has also been linked to reduced parasympathetic activity and increased visceral hypersensitivity, both of which are implicated in bowel dysfunction (43–45).

Beyond behavioral factors, physiological indicators within the LC9 framework, including BMI, glycemic control, lipid levels, and blood pressure, are likewise essential for preserving gastrointestinal health. BMI reflects overall nutritional and metabolic status, which closely correlates with bowel health. Obesity is linked to slower colonic transit, gut dysbiosis, and chronic low-grade inflammation (46, 47). Conversely, being underweight may indicate malnutrition or muscle loss, which can impair gastrointestinal motility and resilience (48). Chronic hyperglycemia, as seen in diabetes, can damage enteric neurons and impair smooth muscle function, leading to diabetic enteropathy. This often manifests as constipation, diarrhea, or both. Glycemic regulation is thus vital for maintaining gastrointestinal neuromuscular coordination (49, 50). Dyslipidemia has been associated with gut microbial imbalance, systemic inflammation, and vascular dysfunction. Altered lipid profiles may reduce gastrointestinal perfusion and mucosal turnover, thereby increasing susceptibility to bowel symptoms (51, 52). Blood pressure regulation influences gastrointestinal health via its effects on vascular tone and intestinal microcirculation. Hypertension may reduce mucosal perfusion, impair nutrient absorption, and contribute to epithelial injury, all of which may compromise gut function (53, 54). Altogether, these lifestyle and metabolic factors, when optimized collectively as reflected by a higher LC9 score, can provide systemic support for bowel function.

To further validate the stability of our study results, we conducted stratified analysis and sensitivity analysis. Stratified analyses showed that LC9 scores were inversely associated with the risk of constipation and diarrhea in most subgroups. These findings support the robustness and generalizability of the association between overall lifestyle health and bowel function. Notably, all interaction tests for constipation were non-significant, suggesting that the protective association between LC9 and constipation is stable and not substantially modified by population characteristics. For diarrhea, while similar inverse associations with LC9 scores were observed in most subgroups, a significant interaction by race was detected. Specifically, the association remained significant among non-Hispanic white people and non-Hispanic Black people but was attenuated in Mexican American and other Hispanic populations. This finding indicates that racial or ethnic background may partially modify the relationship between healthy lifestyle behaviors and diarrhea risk. Possible explanations may include cultural or genetic differences in diet, microbiome composition, healthcare access, or reporting behaviors across racial/ethnic groups. In the sensitivity analyses, we did not weight the data. Unweighted data represent the actual sample of the survey and are not representative of the entire target population. In the analysis of unweighted data, the LC9 scores still showed a negative correlation with constipation and diarrhea, which further confirmed the reliability of our research findings. Beyond its statistical associations, LC9 may also have potential clinical implications. Although no universally accepted cut-off currently defines a “clinically meaningful” LC9 score, our tertile-based analyses indicated that individuals in the lowest LC9 tertile had a markedly higher risk of both constipation and diarrhea compared with those in the highest tertile. This suggests that particularly low LC9 scores may serve as a clinically relevant indicator of poor bowel health. From a practical perspective, LC9 integrates lifestyle and psychological health into a single metric, making it a potentially useful tool for risk stratification in gastroenterology practice. Patients with lower LC9 scores could be identified as a high-risk group and prioritized for targeted lifestyle modification, mental health evaluation, and preventive counseling. Nevertheless, further longitudinal and interventional studies are warranted to establish optimal thresholds and to confirm the predictive utility of LC9 in clinical settings.

Several noteworthy strengths are presented in our research. First, our results are more reliable and applicable to a wider population since we used a large-scale, national NHANES database. Second, our study employed the LC9 framework, an expansion of the LE8 model that incorporates psychological health as an additional dimension. This enhancement allows for a more comprehensive assessment of individual well-being, encompassing both behavioral and physiological factors that may influence bowel health. Another strength of our study is the comprehensive validation strategy. In the primary analyses based on the NHANES cohort, we performed internal validation by randomly dividing the sample into training and validation sets (7:3 ratio), and further incorporated an independent external cohort of 991 patients from our hospital. The model demonstrated stable discrimination across all datasets, with calibration curves confirming good agreement between predicted and observed outcomes. Importantly, DCA revealed meaningful net clinical benefit: for constipation, within a threshold probability range of 0.15–0.65, and for diarrhea, within 0.18–0.67. These findings support the robustness, generalizability, and potential clinical utility of our model in predicting bowel dysfunction. However, this study also has several limitations. First, much of the data comes from self-reported questionnaires, including bowel habits (frequency and stool form) and lifestyle metrics such as diet, sleep, and physical activity. These self-reported measures are inherently subject to recall error and social desirability bias, which may lead to misclassification. Such misclassification bias could either dilute true associations or generate spurious ones, and therefore the results should be interpreted with caution. Second, information about constipation and diarrhea was derived from the most prevalent defecation status over the past 30 days. We do not yet know whether this 30-day indicator better reflects participants’ status in the long-term. Third, it is possible that unknown confounders still impact the connection between LC9 and constipation or diarrhea, even after accounting for several common variables. Finally, our capacity to determine causation is limited by the cross-sectional design, and the observed associations should be interpreted as correlations rather than causal relationships. Moreover, the possibility of reverse causation cannot be excluded. For instance, individuals with chronic constipation or diarrhea may subsequently alter their dietary intake, physical activity, or sleep patterns, and bowel symptoms are also known to adversely affect psychological well-being through the gut–brain axis. These factors may in turn influence LC9 scores, complicating the directionality of the relationship. Future longitudinal cohort studies are warranted to clarify the temporal and causal links between LC9 and bowel health outcomes.

## 5 Conclusion

In conclusion, our study demonstrated that higher LC9 scores were significantly associated with a lower prevalence of both constipation and diarrhea among U.S. adults. By extending the LE8 framework to include psychological health, LC9 offers a more comprehensive evaluation of bowel health. The consistent associations observed across most population subgroups support the robustness and potential clinical relevance of LC9 in bowel health assessment. To corroborate these results, future longitudinal cohort studies are needed to assess the association between LC9 and the development of constipation and diarrhea.

## Data Availability

The raw data supporting the conclusions of this article will be made available by the authors, without undue reservation.
