# Supplementary material for: Association between Life’s Crucial 9 and bowel health among US adults: a cross-sectional analysis of NHANES 2005–2010 with external validation
Source: Front Med (Lausanne). 2025 Oct 31;12:1687499. doi: 10.3389/fmed.2025.1687499 (PMC12615456; doi:10.3389/fmed.2025.1687499)
Supplement: Supplementary file 1 [file Data_Sheet_1.docx]

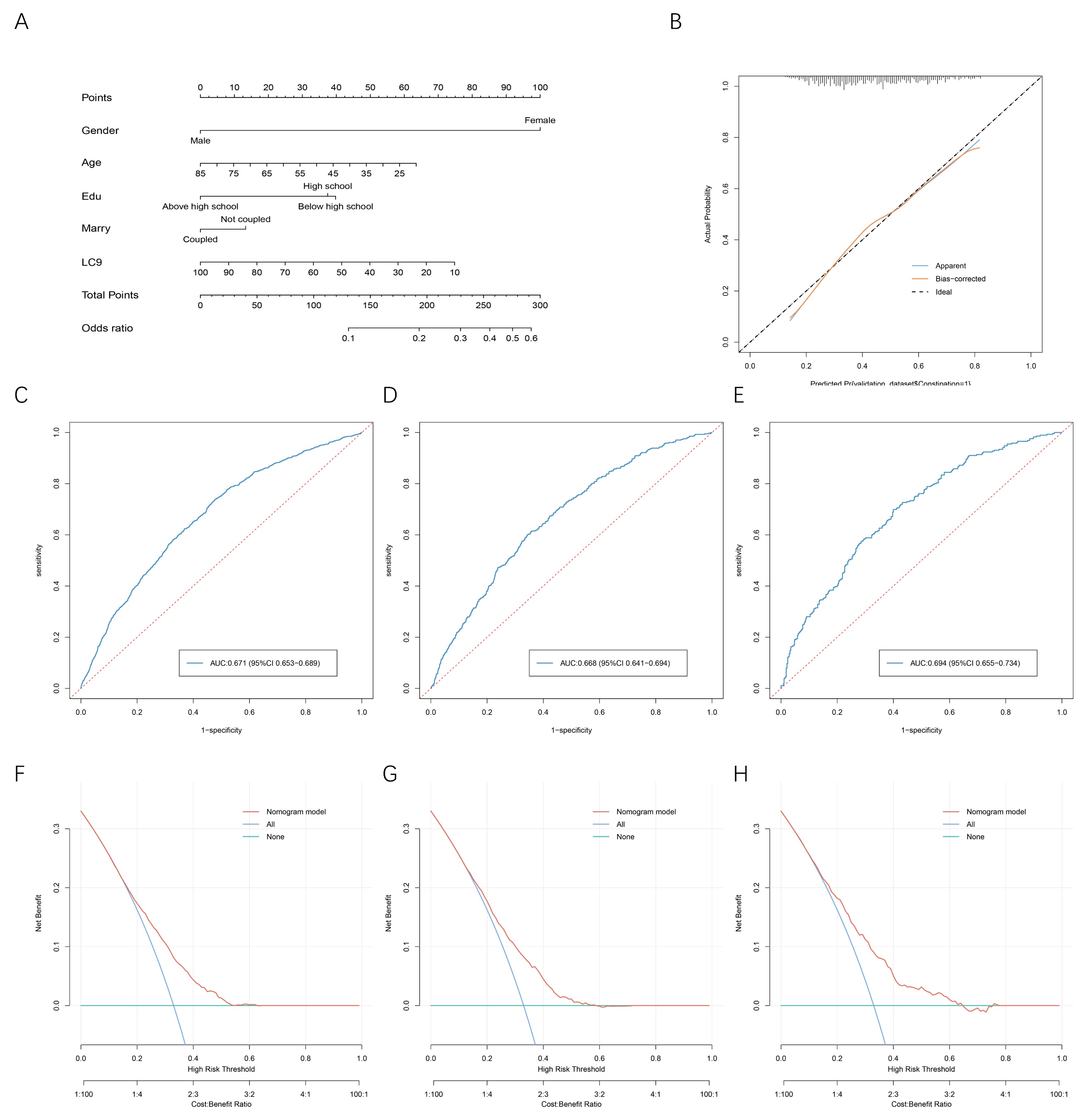


**Supplementary Figure 1.** Nomogram and model validation for predicting constipation based on LC9 and related factors.(A) nomogram constructed based on LC9 and related factors for predicting constipation.(B) calibration curve of the model in the external validation dataset, demonstrating the agreement between predicted probabilities and actual observations.(C-E) ROC curves for the training set, internal validation set, and external validation set, respectively, highlighting the model's predictive performance and discriminatory ability across different datasets. (F-H) DCA for the training set, internal validation set, and external validation set, respectively, evaluating the model's predictive performance and practical value. LC9, Life's Crucial 9; ROC, Receiver Operating Characteristic; AUC, Area Under the Curve; DCA, Decision curve analysis.


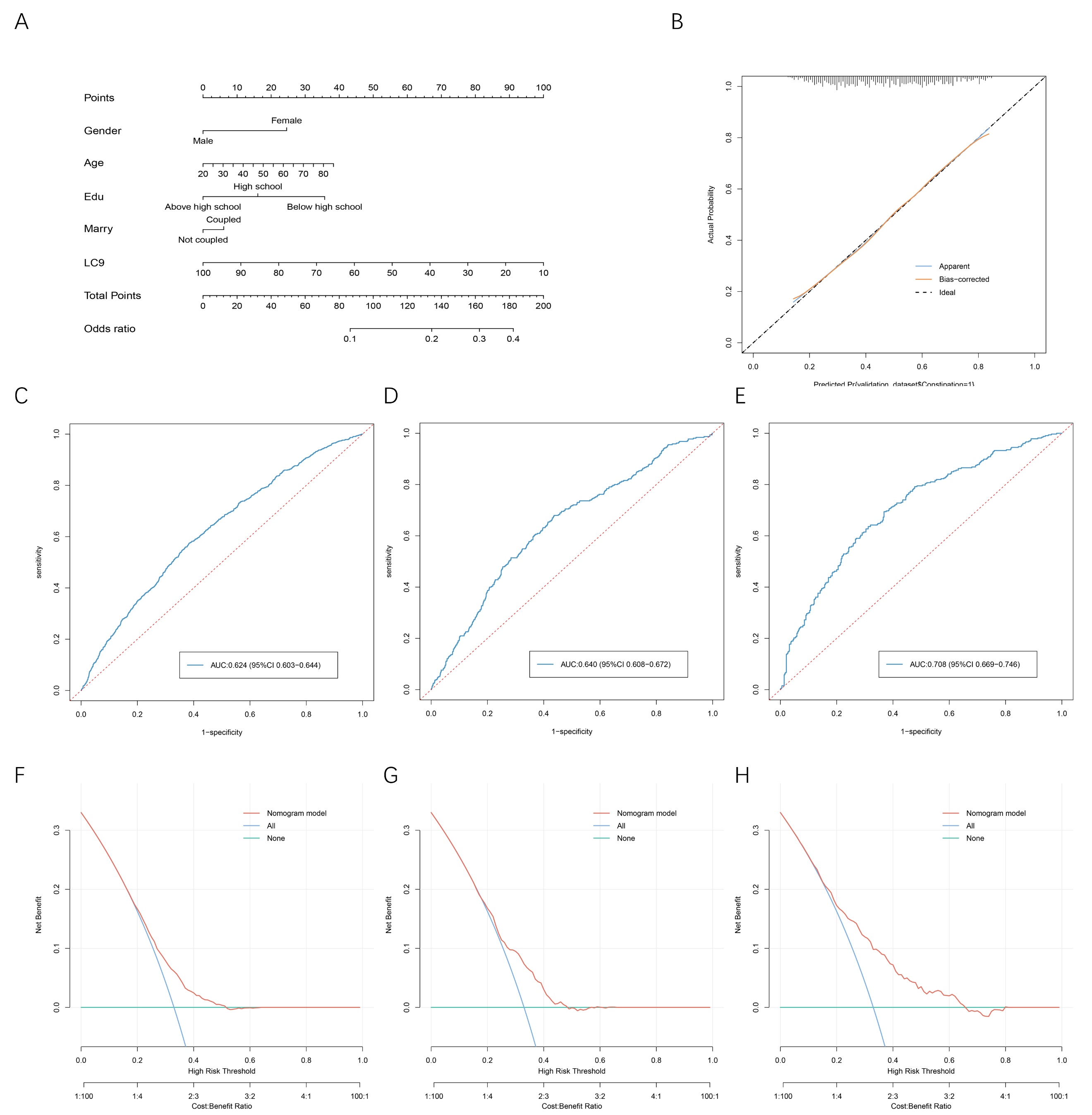


**Supplementary Figure 2.** Nomogram and model validation for predicting diarrhea based on LC9 and related factors.(A) nomogram constructed based on LC9 and related factors for predicting diarrhea.(B) calibration curve of the model in the external validation dataset, demonstrating the agreement between predicted probabilities and actual observations.(C-E) ROC curves for the training set, internal validation set, and external validation set, respectively, highlighting the model's predictive performance and discriminatory ability across different datasets. (F-H) DCA for the training set, internal validation set, and external validation set, respectively, evaluating the model's predictive performance and practical value. LC9, Life's Crucial 9; ROC, Receiver Operating Characteristic; AUC, Area Under the Curve; DCA, Decision curve analysis.
