# Supplementary material for: Association between Life’s Crucial 9 and bowel health among US adults: a cross-sectional analysis of NHANES 2005–2010 with external validation
Source: Front Med (Lausanne). 2025 Oct 31;12:1687499. doi: 10.3389/fmed.2025.1687499 (PMC12615456; doi:10.3389/fmed.2025.1687499)
Supplement: Supplementary file 2 [file Data_Sheet_2.docx]

**Supplementary Table 1. Components and Scoring Criteria of the Healthy Eating Index–2015 (HEI-2015)^*^**

| Component | Maximum points | Standard for maximum score | Standard for minimum score of zero |
| --- | --- | --- | --- |
| Adequacy^#^ | | | |
| Total Fruits[^1^](https://epi.grants.cancer.gov/hei/developing.html#f2) | 5 | ≥0.8 cup equiv. per 1,000 kcal | No Fruit |
| Whole Fruits[^2^](https://epi.grants.cancer.gov/hei/developing.html#f3) | 5 | ≥0.4 cup equiv. per 1,000 kcal | No Whole Fruit |
| Total Vegetables[^3^](https://epi.grants.cancer.gov/hei/developing.html#f4) | 5 | ≥1.1 cup equiv. per 1,000 kcal | No Vegetables |
| Greens and Beans[^3^](https://epi.grants.cancer.gov/hei/developing.html#f4) | 5 | ≥0.2 cup equiv. per 1,000 kcal | No Dark Green Vegetables or Legumes |
| Whole Grains | 10 | ≥1.5 oz equiv. per 1,000 kcal | No Whole Grains |
| Dairy[^4^](https://epi.grants.cancer.gov/hei/developing.html#f5) | 10 | ≥1.3 cup equiv. per 1,000 kcal | No Dairy |
| Total Protein Foods[^5^](https://epi.grants.cancer.gov/hei/developing.html#f6) | 5 | ≥2.5 oz equiv. per 1,000 kcal | No Protein Foods |
| Seafood and Plant Proteins[^5^](https://epi.grants.cancer.gov/hei/developing.html#f6)^,^[^6^](https://epi.grants.cancer.gov/hei/developing.html#f7) | 5 | ≥0.8 oz equiv. per 1,000 kcal | No Seafood or Plant Proteins |
| Fatty Acids[^7^](https://epi.grants.cancer.gov/hei/developing.html#f8) | 10 | (PUFAs + MUFAs)/SFAs ≥2.5 | (PUFAs + MUFAs)/SFAs ≤1.2 |
| Moderation^$^ | | | |
| Refined Grains | 10 | ≤1.8 oz equiv. per 1,000 kcal | ≥4.3 oz equiv. per 1,000 kcal |
| Sodium | 10 | ≤1.1 gram per 1,000 kcal | ≥2.0 grams per 1,000 kcal |
| Added Sugars | 10 | ≤6.5% of energy | ≥26% of energy |
| Saturated Fats | 10 | ≤8% of energy | ≥16% of energy |

**^*^** Intakes between the minimum and maximum standards are scored proportionately.

^#^Adequacy components represent the food groups, subgroups, and dietary elements that are encouraged. For these components, higher scores reflect higher intakes, because higher intakes are desirable.

^$^Moderation components represent the food groups and dietary elements for which there are recommended limits to consumption. For moderation components, higher scores reflect lower intakes, because lower intakes are more desirable.

(1) Includes 100% fruit juice; (2) Includes all forms except juice; (3) Includes legumes (beans and peas); (4) Includes all milk products, such as fluid milk, yogurt, and cheese, and fortified soy beverages; (5) Includes legumes (beans and peas); (6) Includes seafood, nuts, seeds, soy products (other than beverages), and legumes (beans and peas); (7) Ratio of poly- and monounsaturated fatty acids (PUFAs and MUFAs) to saturated fatty acids (SFAs).

**Supplementary Table 2. Overview of the Life’s Crucial 9 (LC9) Score: Components and Scoring Guidelines**

|  | **Health indicators** | **Measurement** | **Quantification and Scoring of Health indicators** |
| --- | --- | --- | --- |
| Psychological  Health | Psychological  Health | Depression Screener Questionnaire (DPQ) | **Metric:** Nine-item depression screening instrument PHQ-9. Each symptom item in PHQ-9 is scored on a 4-point scale, from 0 (‘not at all’) to 3 (‘nearly every day’), resulting in a total score of 0 to 27 points.  **Scoring:**  Points Level  100 the score of 0 to 4 points  75 the score of 5 to 9 points  50 the score of 10 to 14 points  25 the score of 15 to 19 points  0 the score of 20 to 27 points |
| Health Behaviors | Diet | Healthy Eating Index-2015 diet score percentile | Quantiles of DASH-style diet adherence  **Scoring (Population):**  Points Quantile  100 ≥95^th^ percentile (top/ideal diet)  80 75^th^ – 94^th^ percentile  50 50^th^ – 74^th^ percentile  25 25^th^ – 49^th^ percentile  0 1^st^ – 24^th^ percentile (bottom/least ideal quartile) |
|  | Physical activity | Self-reported minutes of moderate or vigorous physical activity per week | **Metric:** Minutes of moderate (or greater) intensity activity per week  **Scoring:**  Points Minutes  100 ≥150  90 120 – 149  80 90 – 119  60 60 – 89  40 30 – 59  20 1 – 29  0 0 |
|  | Nicotine exposure | Self-reported use of cigarettes or inhaled nicotine- delivery system | **Metric:** Combustible tobacco use and/or inhaled NDS use; or secondhand smoke exposure  **Scoring:**  Points Status  100 Never smoker  75 Former smoker, quit ≥5 yrs  50 Former smoker, quit 1 - <5 yrs  25 Former smoker, quit <1 year, or currently using inhaled NDS  0 Current smoker  Subtract 20 points (unless score is 0) for living with active indoor smoker in home |
|  | Sleep health | Self-reported average hours of sleep per night | **Metric:** Average hours of sleep per night  **Scoring:**  Points Level  100 7 – <9  90 9 – <10  70 6 – <7  40 5 – <6 or ≥10  20 4 – <5  0 <4 |
| Health Factors | Body mass index | Body weight (kg) divided by height squared (m^2^) | **Metric:** Body mass index (kg/m^2^)  **Scoring:** Points Level 100 <25  70 25.0 – 29.9  30 30.0 – 34.9  15 35.0 – 39.9  0 ≥40.0 |
|  | Blood lipids | Plasma total and HDL-cholesterol with calculation of non-HDL-cholesterol | **Metric:** Non-HDL-cholesterol (mg/dL)  **Scoring:**  Points Level  100 <130  60 130 – 159  40 160 – 189  20 190 – 219  0 ≥220  If drug-treated level, subtract 20 points |
|  | Blood glucose | Fasting blood glucose or casual hemoglobin A1c | **Metric:** Fasting blood glucose (mg/dL) or Hemoglobin A1c (%)  **Scoring:**  Points Level  100 No history of diabetes and FBG <100 (or HbA1c < 5.7)  60 No diabetes and FBG 100 – 125 (or HbA1c 5.7-6.4) (Pre-diabetes)  40 Diabetes with HbA1c <7.0  30 Diabetes with HbA1c 7.0 – 7.9  20 Diabetes with HbA1c 8.0 – 8.9  10 Diabetes with Hb A1c 9.0 – 9.9  0 Diabetes with HbA1c ≥10.0 |
|  | Blood pressure | Appropriately measured systolic and diastolic blood pressure | **Metric:** Systolic and diastolic blood pressure (mm Hg)  **Scoring:**  Points Level  100 <120/<80 (Optimal)  75 120-129/<80 (Elevated)  50 130-139 or 80-89 (Stage I HTN)  25 140-159 or 90-99  0 ≥160 or ≥100  Subtract 20 points if treated level |

**Supplementary Table 3. Baseline characteristics of the external validation cohort**

| **Variables** | **Constipation (n=289)** | **Diarrhea (n=327)** | **Normal (n=375)** | ***p*** |
| --- | --- | --- | --- | --- |
| Age (years), Mean±SD | 47.56±10.61 | 47.92±11.53 | 43.94±9.96 | <0.001 |
| Sex, n (%) |  |  |  | 0.057 |
| Male | 124 (42.9) | 148 (45.3) | 194 (51.7) |  |
| Female | 165 (57.1) | 179 (54.7) | 181 (48.3) |  |
| Educational level, n (%) |  |  |  | <0.001 |
| Below high school | 66 (22.8) | 73 (22.3) | 56 (14.9) |  |
| High school | 81 (28.0) | 101 (30.9) | 88 (23.5) |  |
| Above high school | 142 (49.1) | 153 (46.8) | 231 (61.6) |  |
| Marital status, n (%) |  |  |  | 0.001 |
| Coupled | 166 (57.4) | 188 (57.5) | 260 (69.3) |  |
| Not coupled | 123 (42.6) | 139 (42.5) | 115 (30.7) |  |
| LC9 score, Mean±SD | 43.68±16.27 | 42.74±15.83 | 52.32±18.26 | <0.001 |
